# On Publication Strategies. Another Advice to a Beginning Scientist

**DOI:** 10.3389/fphys.2020.01073

**Published:** 2020-09-04

**Authors:** Ruben Coronel

**Affiliations:** Department of Experimental Cardiology, Academic Medical Center, University of Amsterdam, Amsterdam, Netherlands

**Keywords:** publication, strategy, controversy, competition, consensus, confirmation

## Abstract

Productivity in science has increased and it is becoming more important for scientists to publish, to publish frequently, and to accumulate citations to their work. However, the peer review system may not only promote and advance but also hinder, prevent, or delay publication. In this personal perspective, confirmatory, consensual, competitive, and controversial publication strategies are described that they may meet with various degrees of approval or disapproval from the author’s peers. The selected publication strategy may impact on the development of a career. Resolving controversies helps science advance efficiently. Therefore, controversies should be sought and addressed, although preferably not at the start of a career.

## Introduction

**Figure 1:**
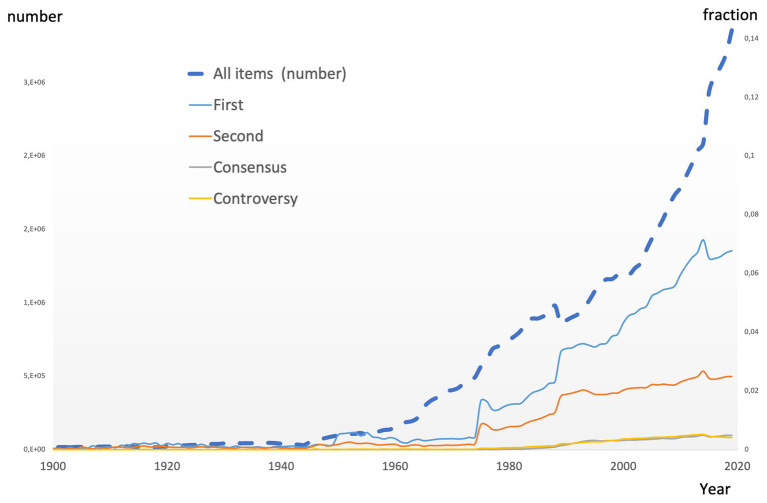
Dotted line: time course of the number of publications (all items) since 1990 (left axis). Source: Web of Science. Drawn lines indicate the number of publications (source: PubMed, search items “first,” “second,” “consensus,” and “controversy”) mentioning “first” (blue), “second” (orange), “consensus” (gray), or “controversy” (yellow) as a fraction of the total number of publications (“all items,” right axis).

The productivity in science has increased logarithmically over the past decades. Figure 1 (bold dotted line) shows the number of published “items” (articles, editorials, and abstracts) since the year 1900 (Opthof and Coronel, 2002). It shows an almost logarithmic increase in the number of published papers starting in the 1960s. Despite this increase in available knowledge, many papers remain uncited (Opthof and Coronel, 2002) and most papers are only cited during the first 2 years after publication (Opthof and Coronel, 2000).

The mass of scientific information that has come available until now and the relatively short “cited” life of the majority of papers may dissuade some scientists to contribute new knowledge. Yet, it has become very important to publish, to publish regularly, and to obtain citations. This is particularly pertinent for biomedical research and physiology. Both fields are characterized by their high potential impact on society but also by the fierce competition between its workers for the available funding. To publish regularly is a sign of active involvement in research and helps in the acquisition of funding. In addition, being cited is considered an indication of scientific quality. Publication of scientific reports can be helped, promoted but also hindered, delayed, or even prevented by one’s peers. Scientists, especially when they are at the start of their career, may want to spend some thoughts on their publication strategy and on how to be successful in publishing. In the below personal thoughts, I will offer some unrequested and likely unwanted advice, like others before me (Medawar, 1979).

## Peer Review

Eventual publication of the scientific reports depends on the gatekeeper function of the peer review system. This selection procedure stems from a time when printed pages were expensive and only a limited amount of papers could be printed. Hence, only the best papers could be accepted and a quality assessment by several of the author’s peers was the preferred selection method. Various forms of peer review exist, but they all involve this assessment of the scientific report by peers. In other sections of science, other forms of peer review are used whereby a paper can appear in a “pre-print” version and to which the entire community can add their comments.

In biomedical sciences and physiology, the author’s peers are often also competitors for the same funding or contestant in the race of publishing data first. Authors and their peers, therefore, form a social network in which publication of the results of scientific research is regulated. A new member of the group is admitted by agreement of the existing members (co-optation). This has implications. One is that a scientist has to please the network and earn his/her place in the network before he or she can challenge it. Another one is that the allowed increment of scientific knowledge in scientific publications is limited, because the author is not permitted to exceed the limits of the allowed “niche” and because it is in the interest of the network to spread the new information over as many publications as possible. Because a similar peer review system is operative for the distribution of funding of research, failure to publish, therefore, often implies the failure to acquire funding. It is, therefore, important for a – beginning – scientist to think about a publication strategy before publishing a highly competitive or controversial paper. One strategy is to defer a controversial publication until the social network of peers is “primed.”

Awareness of various publication types may help the – young – scientist to choose a publication strategy and to eventually obtain a position in which he/she is respected and able to publish even the most controversial ideas. Each of the following four publication types can be equally valuable and helpful for the advancement of science, but they differ in their effect on the readership and peers. As with all classification system, combinations of these classes occur and other classifications are possible.

## Confirmatory “Consumerist” Publication Strategy

The consumers of a scientific publication are represented by the readership and the peer reviewers. It is in the interest of these consumers to be informed of research that does encroach on their own field of research and that does not challenge the existing scientific ideas too much. A consumerist, confirmatory, publication will thus maintain the existing scientific *status quo* and will not overhaul existing systems of distribution of funding. It is friendly to the peers and it can be conceived as “doing what is expected.”

A confirmatory publication is characterized by testing an existing hypothesis, by expanding an existing hypothesis in a reductionist or integrative fashion (applying it on a smaller or larger scale), by application of this hypothesis to other species/models, or by describing associated phenomena without an overruling new hypothesis. I surmise that the majority of publications are of the confirmatory consumerist type.

My own 1988 publication (my first as a first author) about the change of extracellular potassium concentration during acute myocardial ischemia can be seen as a confirmatory publication (Coronel et al., 1988). It built upon an existing idea of heterogeneous increase of extracellular potassium concentration in myocardial ischemia (Hill and Gettes, 1980) and its influence on the genesis of life-threatening arrhythmias (Kléber et al., 1987) and, thus, extended an existing idea. Nevertheless, it still is being cited from time to time.

## Consensual Publication Strategy

Consensus reports are often published to guide clinical practice and to standardize diagnosis and/or treatment of patients. Scientists from various countries and disciplines often confer and reach consensus about a disease, including its pathophysiological mechanisms. Even when there is disagreement about some aspects of the mechanism or about treatment, an effort is made to reach consensus or to reach consensus on which points to disagree. Consensus is often reached by voting on various theses and, therefore, involves a democratic (co-optational) process. The ensuing report is often published in various journals at the same time in order to emphasize the broad – network wide – support of the agreement reached. A consensual publication is an expression of the idea that scientific research is a group effort and, thus, supports the notion that the scientific community is a social network.

Usually, a consensual publication is typified by a large number of authors, most of whom are authorities on the topic of interest. The paper usually contains a review of the literature and includes citations to publications by each of the co-authors. The publication is often supported or endorsed by one or more international professional societies. Other types of consensual publications occur when authors agree to publish on the same subject and explore their differences. They agree to disagree.

Examples of consensus reports are the ones on Brugada syndrome, a potentially lethal syndrome occurring in relatively young patients (Antzelevitch et al., 2005, 2017). The publications were updated several times as an expression of the changing ideas regarding diagnosis, treatment, and the pathophysiological mechanism. An example of a publication in which the authors agree to disagree is a paper about the potential mechanisms of Brugada syndrome (Wilde et al., 2010).

Figure 1 (gray line) shows the percentage of consensus papers (mentioning “consensus”) relative to the total amount of papers in the same year of publication. There is a small relative increase in consensus papers since the 1990s, but the volume remains very low (about 0.5%; source PubMed).

## Competitive Publication Strategy

A competitive publication strategy often involves the question who is first to publish in a competitive field of research. Contrary to consensual publication (see above), it emphasizes the idea that an individual or a single group can stand out and excel in science. Indeed, a single paper may change the way we think about a subject and the first person to publish it can acquire eternal fame. The publication that comes second is then considered confirmative and not as ground breaking as the first. A publication of the competitive type usually contains a claim that the authors are the first to describe the particular scientific content, often in the summary, in the introduction, or in the discussion: “*This is (to the best of our knowledge) the first (description of)…*” (Cui et al., 2020; Docrat et al., 2020).

An example of competitive publication is the work by Watson and Crick (1953) on the structure of DNA, with which they became famous. Although these authors did not mention the word “first” in their text, their modest phrasing of the implication of their work is an understatement that brings home the message that they were the very first to see the importance of their work: “*It has not escaped our notice that the specific pairing we have postulated immediately suggests a possible copying mechanism for the genetic material*” (Watson and Crick, 1953).

Figure 1 (blue line) shows the number of publications containing the word “first” relative to the total number of publications in the same year. It shows that “first” increasingly occurs in scientific texts since the 1970s, even if corrected for the increased productivity, and that it reaches about 8% of the total. Of course, it cannot be excluded that “first” was not always used as a claim of novelty (“first child” and “first myocardial infarction”). Therefore, the relative occurrence of “second” (orange line) in publications is also shown. It reaches much lower than “first” (reaching 2.5%). It remains to be determined whether a publication containing the claim to be the first is justified and is also cited more often than other papers.

## Controversial Publication Strategy

Controversy in science is often associated with strong emotions of consent or disapproval. Although a scientist ideally is disinterested in the outcome of research, he/she also is human. A change of a way of thinking and a different interpretation of what is the truth can be unsettling, especially if one has spent a lifetime on the now disputed truth. Planck has expressed this somewhat pessimistic view on how scientists deal with changed paradigms: “*A new scientific truth does not triumph by convincing its opponents and making them see the light, but rather because its opponents eventually die, and a new generation grows up that is familiar with it*” (Planck, 1950). Even though addressing controversies is an efficient and quick route to scientific advancement, their publication is cumbersome. First, publishing controversial papers is difficult because the peer review system is essentially conservative and will allow new ideas only if the traditional view is explored in depth first. This will complicate and slow the peer review process. Submitted controversial papers can be rejected at various journals but typically undergo multiple revisions (if these are allowed). Second, if the paper is published, it will encounter adverse reactions ranging from unwillingness to cite the paper or use of contrary arguments without applying the concept of scientific discourse and “fair hearing.” Therefore, a controversial paper is usually tainted by the results of a long peer review process and contains multiple lines of evidence, a long discussion and extensive citation to work of the opponents.

The delayed publication (“Darwin’s delay”) of the highly controversial “On the origin of species” and the separate publication of the “The descent of man” by Darwin may be examples of a publication strategy in which Darwin let his peers become acquainted with his ideas on evolution prior to extrapolating these ideas to the human species (Darwin, 1859, 1871). Other examples of controversial publications occur in my own publication list (Coronel et al., 2005; Janse et al., 2011, 2012).

A special case is formed by papers in which a failure to replicate earlier findings is published. These papers are often associated with the same strong emotions and eventually may lead to controversy, if the difference is not resolved. Publication of the “negative results” is difficult, because the exclusion of all alternative explanations has provided. Publications of this type are rare, although probably very valuable to bring science forward.

Figure 1 (yellow line) shows the percentage of controversial papers (mentioning “controversy”) relative to the total amount of papers in the same year of publication. There is a small relative increase in controversy papers but its volume remains very low (about 0.5% of publications). The observation that its occurrence is about the same as that of “consensus” papers is maybe not surprising if one considers that consensus is often required when there is controversy.

## Publication Strategies and Career Planning

If we accept that the scientific community is a social network in which co-optation plays a role, acceptance of the scientist by his/her group of peers is important for his/her professional career. Because publication success is dependent of the gatekeepers of the peer review process, scientists should think about how to publish their research before embarking on the study. In the above, I have mentioned four possible – arbitrary – publication modalities with increasing chance of confrontation with the peer group. Of course, more classifications of publication modes exist, and the categories mentioned are not mutually exclusive. It is not easy to define whether a particular publication type is better for a career than another. If a controversy can be resolved (within a life-time, see the quote from Planck) or a major advance is published in a competitive paper, it may bring personal success and a favorable position for acquiring funding to the authors of the paper. However, this may take more time than is permitted for a scientist during his/her PhD-trajectory (usually 3–4 years), during a post-doctoral fellowship (usually 2 years), or for junior faculty. These beginning scientists may consider to concentrate on confirmatory or consensual publications in their own research, while participating in research groups to obtain more “risky” publications (competitive and controversial). Publishing a controversial paper as a first author and as a first publication may be disadvantageous. It should rather be recommendable to publish a confirmatory or a consensual paper before a competitive or controversial paper and entering into battle with the peers. Postponing the battle until enough critical mass – support from others – has been acquired may be preferred for a starting career.

The advantages and disadvantages of the various publication strategies thus depend on the peer review system and we should consider changing this widely accepted system of quality assessment. However, changing it into other forms (open peer review, blinded peer review, and prepublication archives) will not change the essential characteristic of the scientific world as a social network where co-optation is the norm. Open peer review, as practiced by Frontiers, increases transparency about the potential biases of the peers, because the names of the peer reviewers are disclosed but does not change the essential method of co-optation. Controversial publications bring science forward most efficiently. Publishing houses may promote controversial publications and offer platforms for public debate (Lakatta and DiFrancesco, 2009; Efimov et al., 2010).

### Conclusion

Consensus in the form of a compromise is generally not good for science, because truth usually is not amenable to bartering. If a controversy exists, consensus may be reached on what to agree on and on what to disagree on. Eventually, resolving controversies helps science advance efficiently. Therefore, scientific controversies should be sought and resolved, and their publication promoted.

## Data Availability Statement

The original contributions presented in the study are included in the article/supplementary material, further inquiries can be directed to the corresponding author.

## Author Contributions

The author confirms being the sole contributor of this work and has approved it for publication.

### Conflict of Interest

The author declares that the research was conducted in the absence of any commercial or financial relationships that could be construed as a potential conflict of interest.
